# Cerebellin-1 leads the way

**DOI:** 10.1371/journal.pbio.3001880

**Published:** 2022-11-18

**Authors:** Laura E. McCormick, Stephanie L. Gupton

**Affiliations:** University of North Carolina at Chapel Hill, Chapel Hill, North Carolina, United States of America

## Abstract

The cerebellin family of proteins influences synapse formation and function. In this issue of *PLOS Biology*, Han and colleagues identify a new role for Cerebellin-1 in axon growth and guidance.

In development, neurons are faced with an immense challenge. As neurons grow and develop their intricate, complex shapes, they must also grow in the appropriate directions. In particular, their thin, elongated axon can travel large distances to connect with postsynaptic partners. In the past decades, the scientific community has identified an expansive catalogue of attractive and repulsive axon guidance cues that serve as road signs for the growing axon. The nature of the cues varies greatly at the molecular level—some act as soluble, chemotactic cues, while others act as adhesive, haptotactic cues. Often amplified by differentially expressed extracellular receptors and coreceptors, this vast collection of guidance cues enables different axons to navigate to precise and distinct positions in a neuron subtype-specific way.

Interestingly, in recent years, a number of proteins and pathways involved in axon guidance have also been shown to be involved in the developmentally distinct process of synaptogenesis [1]. This repurposing of pathways in multiple stages of development allows a limited number of genes to have multifaceted functions.

Cerebellin-1 (Cbln1) was first described as a glycoprotein secreted in the cerebellum where it is required for proper synaptic function between parallel fibers and Purkinje cells [2]. Notably, Cbln1 acts *trans*-synaptically, binding both presynaptic neurexins and postsynaptic GluD2 [3]. Although Cbln1 was originally thought to only play a role in the cerebellum, more recent work demonstrates that the cerebellin proteins are expressed in a variety of brain regions [4,5]. For example, Cbln1 influences synapse formation in cortical and hippocampal cells [6]. However, whether Cbln1 played a role during neuronal development outside of synapse organization is still unknown.

In this issue of *PLOS Biology*, Han and colleagues report a new role for Cbln1 in axon growth and guidance [7] (Fig 1). The authors first found that Cbln1 is expressed at early stages of murine spinal cord development, well before synaptogenesis. Although Cbln1 was expressed in the spinal cord floor plate at embryonic day 10.5 (E10.5), relatively little Cbln1 was observed in the dorsal commissural neurons (DCNs) that project from one side of the spinal cord to the other. Strikingly though, they observed a rapid increase in Cbln1 expression in the DCNs from E10.5 to E12.5, overlapping with midline crossing.

**Fig 1:**
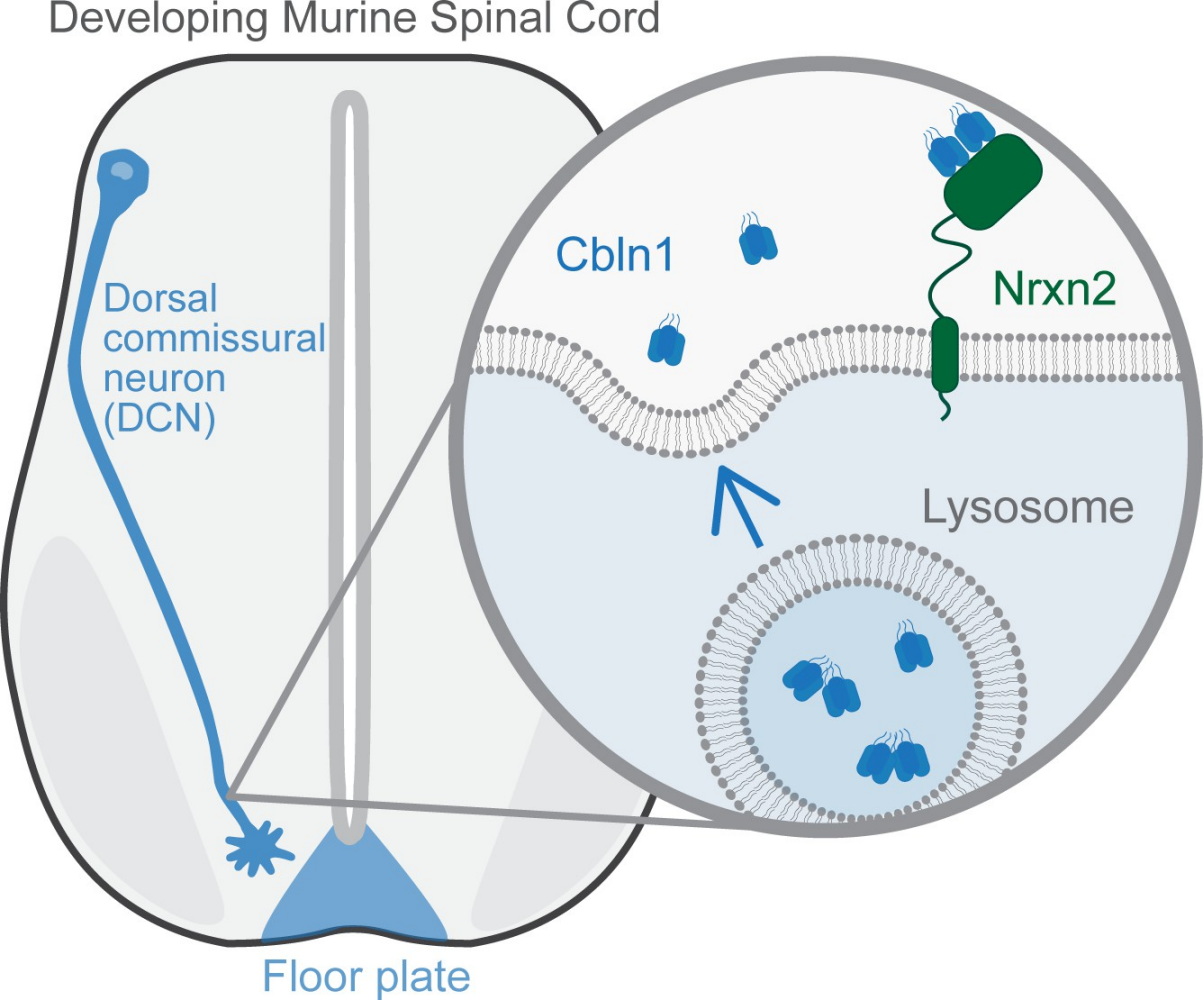
Cbln1, secreted by both the floor plate and DCNs, acts as an attractive axon guidance cue during spinal cord development. In DCNs, Cbln1 is released through lysosomal exocytosis and binds to its extracellular receptor Nrxn2. Cbln1, Cerebellin-1; DCN, dorsal commissural neuron; Nrxn2, Neurexin-2.

As this expression pattern suggested a potential role for Cbln1 in axon guidance, the authors used a conditional knockout (cKO) mouse to delete Cbln1 specifically in spinal cord DCNs. In vivo, DCNs lacking *Cbln1* showed impeded axonal growth. Similar decreases in axon length and number were observed in *Cbln1* cKO DCN explants. Critically, the addition of recombinant Cbln1 protein to culture media rescued these stunted axonal phenotypes.

Consistent with previous studies in cerebellar granule cells [8], the authors confirmed that Cbln1 is secreted from the DCNs through lysosomal exocytosis. As synaptic binding partners of Cbln1 are known [3], the authors hypothesized that these binding partners and downstream signaling pathways were conserved in their newly identified function in axon guidance. They determined that the receptor Neurexin-2 (Nrxn2) is expressed in the axons and growth cones of DCNs. Furthermore, the presence of Nrxn2 is required for the axon outgrowth of cultured DCNs toward Cbln1-expressing cells.

In parallel, the authors also focused on the role of Cbln1 in the floor plate—could secreted Cbln1 from this structure influence DCN growth? Upon creating a floor plate–specific *Cbln1* cKO mouse, axon guidance deficits were once again observed. In particular, the ventral commissure—the bundle of axons crossing the midline—was thinner and changed in shape.

Having identified a new role of Cbln1 in spinal cord axon guidance, Han and colleagues also examined axonal growth and guidance in other brain structures. Returning to the roots of Cbln1 research, the authors explored Cbln1 expression patterns in the cerebellum. Similar to the spinal cord, Cbln1 was expressed at early time points and was required for proper axon outgrowth from cerebellar granule cells. Finally, the authors focused on the optic chiasm. Interestingly, retinal ganglion cells do not express Cbln1, but nearby cells in the ventral diencephalon do. Akin to the spinal floor plate, the secretion of Cbln1 from the diencephalon influenced the guidance of retinal ganglion projections.

This research highlights an exciting fundamental role for Cbln1 in axon growth and guidance that is conserved in multiple regions of the brain. With this precedent, future work is warranted to determine if Cbln1 plays a role in additional midline crossing events—such as the formation of the corpus callosum in the cortex. Likewise, synergy between different pathways fine-tunes axon pathfinding. Whether coordination exists between Cbln1 and other guidance cues—whether they be attractive or repulsive—is an open question.

Of note, Cbln1 is just 1 protein in the 4-member cerebellin family. Interestingly, Cbln1 and Cbln3 form mixed multimers and are secreted together from cerebellar granule cells [9], suggesting that Cbln3 could also play a role in axon guidance.

Furthermore, this work also highlights the dual roles of Cbln1-mediated axon growth and guidance—cell autonomous and noncell autonomous—as both DCN-secreted and floor plate–secreted Cbln1 influence DCN pathfinding. However, it will be interesting to test if both secreted forms of Cbln1 play distinct roles in axon growth and guidance, perhaps varying in their splicing or posttranslational modifications? Similarly, whether Cbln1 acts as a soluble chemotactic cue or adhesive haptotactic cue remains to be defined.

Finally, Cbln1 is identified as another example in which a secreted protein and its extracellular receptor plays a role in both axon guidance and synaptogenesis. However, the authors ponder if synaptic signaling pathways downstream of Cbln1-Nrxn2 are conserved in axon guidance. Similarly, we also question if Cbln1 signaling may influence other early stages of neuronal morphogenesis, such as dendritic arborization. As the list of molecular cues playing a role at multiple developmental time points increases, the field will undoubtedly start testing how these cues can switch between fundamentally distinct processes, yet maintain temporal coordination.

## Abbreviations

Cbln1: Cerebellin-1
cKO: conditional knockout
DCN: dorsal commissural neuron
Nrxn2: Neurexin-2
